# Degradation of dye containing in textile wastewater by sequential process: photocatalytic and biological treatment

**DOI:** 10.55730/1300-0527.3501

**Published:** 2022-09-27

**Authors:** José Carlos MENDOZA HERNÁNDEZ, Gabriela PÉREZ OSORIO, José Eligio Moisés GUTIÉRREZ ARIAS, Josefina CASTAÑEDA CAMACHO

**Affiliations:** 1 Faculty of Chemical Engineering, Meritorious Autonomous University of Puebla, Puebla, Mexico; 2 Faculty of Electronic Sciences, Meritorious Autonomous University of Puebla, Puebla, Mexico

**Keywords:** Alumina, bacterial consortium, erionite, photocatalysis, textile wastewater, toxicity test

## Abstract

In this research, a combined photocatalytic and biological treatment is proposed for the elimination of pollutants present in textile wastewater using a natural erionite zeolite (PE) and aluminum oxide (PA) synthesized by the sol-gel method as photocatalysts, and solar radiation. Both catalysts were characterized by XRD, SEM, and EDS. For biological treatment two bacterial consortium were used: BC1 *(Escherichia coli N16, Serratia k120, Pseudomonas putida B03 and Enterobacter hormaechei*), and consortium BC2 (*Escherichia coli N16, Serratia Mc107, Enterobacter N9, Enterobacter hormaechei Mc9*). The photocatalytic and microbiological treatments were carried out initially separately and subsequently in a sequential manner, first the photocatalytic followed by the microbiological to determine if a synergistic effect was achieved. Comparing the photocatalytic performance, erionite showed higher performance of dyes degradation (54.75%) than alumina (28.62%). While in the biological process, BC1 decreased the dye concentration to 56.93% and BC2 to 53.56%. Finally, the best combined process was PA+BC1 reaching pollutants degradation 64.62%, showing that the application of both processes promotes a decolorization in textile wastewater. The water resulting from the combined photocatalysis-microbiological degradation processes was tested for toxicity using *Daphnia magna*, obtaining that none of the effluents shows toxicity.

## 1. Introduction

The contamination of surface water bodies is a growing environmental problem from diverse sources, such as wastewater discharges from industries, especially those that use colorants. Untreated colored wastewater discharges represent significant damage to aquatic species and balance of aquatic environments. The negative effects of the presence of dyes in water are diverse, from aesthetic such as the presence of turbidity and color to serious on human health such as mutagenic and carcinogenic damage due to bioaccumulation in the food chain. The impact on aquatic environments is mainly in the reduction of light that penetrates the bodies of water, altering the photosynthetic processes, decreasing the concentration of dissolved oxygen, and causing eutrophication. Concerns about discharges from the textile industry are of the greatest concern due to the large volumes of effluents and the fact that the dyes discharged are highly toxic and recalcitrant [1].

Dyes can be classified by their chemical structure or by their affinity and fixation on different objects and substances. In general, they are complex chemical structures that can contain one or more aromatic rings linked to elements such as sulfur and nitrogen or to functional groups that give them very particular characteristics. Such synthetic structures turn out to be highly stable and persistent, so under normal environmental conditions they are difficult to degrade [2].

Advanced oxidation processes, such as heterogeneous photocatalysis, represent a type of emerging technology that aims to eliminate recalcitrant pollutants such as colorants because these can be eliminated or degraded with conventional wastewater treatment methods. Heterogeneous photocatalysis has been widely investigated, using different materials as photocatalysts and with an artificial light source such as UV lamps or natural such as solar radiation. Among the most outstanding advantages are the in situ generation of reactive radicals, mineralization of pollutants, there is no production of sludge. While its main disadvantages are generation of byproducts, difficult scaling for industrial applications, not achieving complete mineralization [3, 4].

A variety of materials has been used as photocatalyst, mainly TiO_2_ single and modified with metals such as Cu, Fe, Zn [5], and other oxides such as ZnO, Al_2_O_3_ [6], SiO_2_ including clays and zeolites [7].

Zeolites are aluminosilicates that have been widely used in industry as catalysts for several decades due to their outstanding characteristics such as high specific surface area, thermal resistance, and cation exchange capacity, among others. In the same way, they have been used as catalyst support in a wide variety of applications in the environmental area for the elimination of pollutants in water and air. Recent research aims at determining the factors that improve the catalytic performance of zeolite-based photocatalysts for the degradation of recalcitrant organic pollutants in water. This depends to a great extent on its adsorption capacity, as well as its efficiency in separating and transferring the photogenerated charges [8].

Alumina has been widely used for several decades as adsorbent, catalyst and catalyst support due to its characteristics such as high thermal and mechanical stability and its high specific surface area. However, it has recently been shown that it also participates and has an important effect on the photocatalytic performance of composites such as CeO_2_/alumina used for the degradation of colorants with visible light [9]. Al_2_O_3_ has been investigated since nanoparticles decrease band gap in comparison to its bulk state, which represents the possibility of being applied as a photocatalyst in the presence of sunlight [10]. Photodegradation of bisphenol A was achieved with a series of alumina-coupled iron oxides, it was demonstrated that dependence of bisphenol degradation on alumina content was attributable to the crystal structure, crystalline and surface structure [11].

The use of microorganisms such as algae, fungi, and bacteria has been investigated in recent years, to discolor, transform or mineralize textile dyes [1]. In the case of bacteria, it has been shown that they can biodegrade several polluting dyes commonly used in the textile industries such as Novacron Super Black G. After 96 h of treatment with bacteria isolated from effluents of textile industries Novacron Super Black G was not completely mineralized and some new metabolites were formed in culture supernatant [12].

Among the challenges to be faced to treat wastewater from the textile industry are evaluating the toxicity of reaction products to ensure that toxic byproducts are not being generated and combining two or more technologies to achieve a higher percentage of degradation [13].

In the present work, a combined sequential photocatalytic-microbiological system for the decolorization of textile wastewater is proposed. It is considered that both systems have the benefits of being friendly to the environment, because the photocatalysis system uses sunlight, while the bioremediation system with bacterial consortia is an easy-to-apply and effective system, the health risks human, animal, or vegetable are minimal and do not require further treatment. The purpose of applying the photocatalytic process first is because the photolysis of the dye molecules begins, leaving less complex chemical structures [14] and easier degradation for the bacteria that will be used in the microbiological process, promoting a synergy for both processes and achieving a greater degradation of textile dyes. The proposed catalysts, one natural mineral and the other synthetic (erionite and aluminum oxide), have not been reported in bleaching treatments with sequential combined microbiological processes. The best combined treatment was PA+BC2 reaching COD reduction of 92% and 91.2% of decolorization, followed by PA+BC1 with 85% and 86.4%, respectively. Meanwhile, the treatment PA+BC1 reached the higher degradation of 64.62% of the total pollutants containing in the textile wastewater. In general, degradation of dye containing in textile wastewater by sequential process: photocatalytic and biological treatment was PA+BC1 > PA+BC2 > PE+BC1 > BC1 > PE+BC2 > PE > BC2 > PA.

## 2. Materials and methods

### 2.1. Photocatalytic degradation by solar radiation

Two materials were used as photocatalysts a natural zeolite type erionite and synthetic aluminum oxide (Al_2_O_3_). Natural zeolite erionite is from Agua Prieta (Sonora, Mexico), this mineral was crushed and sieved to 250 mesh and used without previous treatment. The catalyst Al_2_O_3_ was synthesized by a sol-gel method from organic precursors [15]. A solution of aluminum sec-butoxide (Alfa Aesar) was prepared using 25 mL of 2-methyl-2, 4-pentanediol (Alfa Aesar), and maintained in reflux for 3 h, with moderate agitation at 94 ºC. The gel was made by hydrolysis with deionized water and aged for 10 h, water/alkoxide ratio was 1.388:0.196. Aluminum oxide was dried at vacuum (40 cm Hg) and temperature of 100 ºC for 12 h and then calcined in N_2_ atmosphere at 450 ºC for 12 h, with a later treatment in air at 650 ºC for 4 h. Both catalysts were characterized by X-ray diffraction using a Bruker model D8 Discover diffractometer, scanning electron microscopy and energy dispersive spectroscopy using a microscope JEOL JSM-6610 LV, with an Oxford detector model INCA.

A tubular solar collector was used for photocatalytic test (36 mm in external diameter, 600 mm of length and 0.5 L of volume), it was made of borosilicate which has a 95% UV transmission on high reflectance aluminum sheets (involute). To prevent evaporation of textile wastewater (TWW) and to keep air diffusion and dispersion catalysts, it was coupled to the solar collector a refrigeration system and an air pump (400 mL/min). Then, 45 mg of catalyst was added to 450 mL of TWW and exposed to solar radiation for 6 h (PA: photocatalysis with alumina and PE: photocatalysis with erionite). The solar collector was placed in an area where sunlight received completely in a north-south orientation, with 19 degrees of inclination (Altitude of Puebla city) respect to Ecuador. Experiments were started at 9:00 a.m. and were taken an aliquot of 10 mL each hour to evaluate dye degradation with a UV-Vis spectrophotometer.

### 2.2. Bacterial biodegradation

A consortium composed of three bacterial strains was used: *Escherichia coli N16, Serratia k120, Serratia Mc107, Enterobacter N9* which were previously isolated from mine tailings and characterized as vegetable growth promotors [16]; *Pseudomonas putida B03 and Enterobacter hormaechei Mc9*, which was isolated from a hydrocarbon spilling area in Acatzingo y San Martín Texmelucan, Puebla, Mexico, respectively [17].

The inoculant for the biodegradation of the textile wastewater after photochemical degradation was prepared by individually growing the bacterial strains *Escherichia coli N16, Serratia k120, Serratia Mc107, Pseudomonas putida B03, Enterobacter N9* and *Enterobacter hormaechei Mc9* in Luria Bertani (LB) broth, incubated at 30 °C for 48 h by orbital stirring at 80 rpm. Later, they were separated by centrifugation at 8000 rpm for 20 min.

The biological reactor Tecno-lab MAG 5L was prepared containing 3 L of the photochemical wastewater reaction product to be degraded (PA and PE), 20 g of the bacterial consortium one (BC1) containing 4 g of each strain (*Escherichia coli N16, Serratia k120, Pseudomonas putida B03 and Enterobacter hormaechei*), or consortium two (BC2) containing 4 g of each strain (*Escherichia coli N16, Serratia Mc107, Enterobacter N9, Enterobacter hormaechei Mc9*) and micronutrients (composition per g L^−1^: KH_2_PO_4_ 0.4, K_2_HPO_4_ 2, MgSO_4_ 0.2, CaCl_2_ 0.1, FeSO_4_ 0.005, H_3_BO_3_ 0.002, ZnSO_4_ 0.005, NaMo 0.001, MnSO_4_ 0.003, CoSO_4_ 0.001, CuSO_4_ 0.001, NiSO_4_ 0.001), being incubated at 30 °C by stirring at 80 rpm for 6 days.

The degradation percentage was monitored by taking 3 mL of aliquots every 24 h for 6 days and separating the bacteria by centrifugation at 8000 rpm for 10 min. The biodegradation was monitored by UV-visible spectroscopy.

### 2.3. Physicochemical characterization of textile wastewater

The real textile wastewater was analyzed before and after the combined treatment. Determination of physicochemical parameters were made according to the Official Mexican Standards NMX, pH (NMX-AA-008), total suspended solids (NMX-AA-034), and conductivity (NMX-AA-093). On the other hand, methods 113 and 139 of the Merck SQ 118 spectrophotometer were used to determine turbidity and color. For the determination of the chemical oxygen demand (COD) the photometric method was used with the COD cell test kit (Merck) in a range of 25–1500 mg/L, analyzed in the NOVA 60A spectrophotometer.

### 2.4. Acute toxicity test with Daphnia magna

The acute toxicity test of the degradation products from the three dyes, obtained at the end of the combined treatment system, was carried out by using the methodology stated in Norma Official Mexicana NMX-087-SCFI-2010 for the analysis of acute toxicity in water. Exploratory tests were carried out diluted to 100%, 50%, 25%, 12.5%, and 6.25% of the samples obtained once the bacterial biodegradation ended, by using reconstituted water as a dilution medium and, in case there was a toxicity in the samples, lethal dose 50 (LD50) was calculated by defining trials exposing the organisms to at least five dilutions in the range of observed toxicity. Every liter of reconstituted water was prepared with solutions of 25 mL of dehydrated calcium chloride (11.76 g), magnesium sulfate pentahydrate (4.93 g), sodium bicarbonate (2.59 g), and potassium chloride (0.23 g); all of them diluted in one liter. In each sample container, 30 mL of each one of the prepared diluted solutions were placed and 10 newborn of less than 24 h old were placed. In each case, the trials were carried out in triplicate for each dilution. Additionally, both a positive and a negative control were prepared to test sensitivity and appropriate health status of the sample organisms, respectively. Exposure was 48 h in all cases, doing prereadings at 24 h. These control readings allowed the presence or absence of movement in the reference organisms to be tested.

## 3. Results

### 3.1. XRD

**Figure 1:**
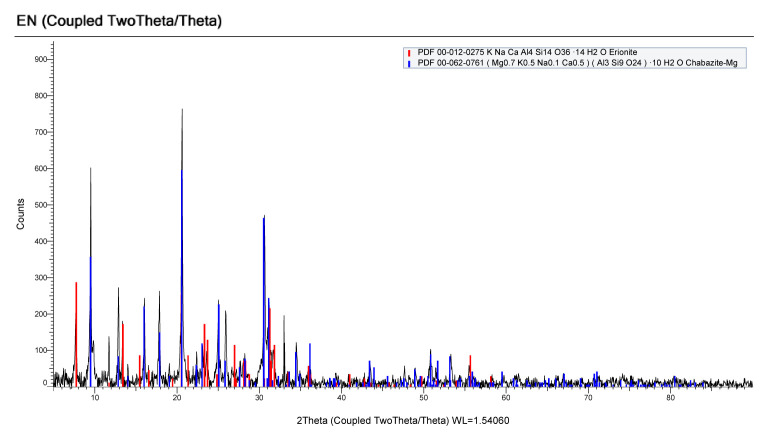
XRD pattern of natural zeolite erionite.

**Figure 2:**
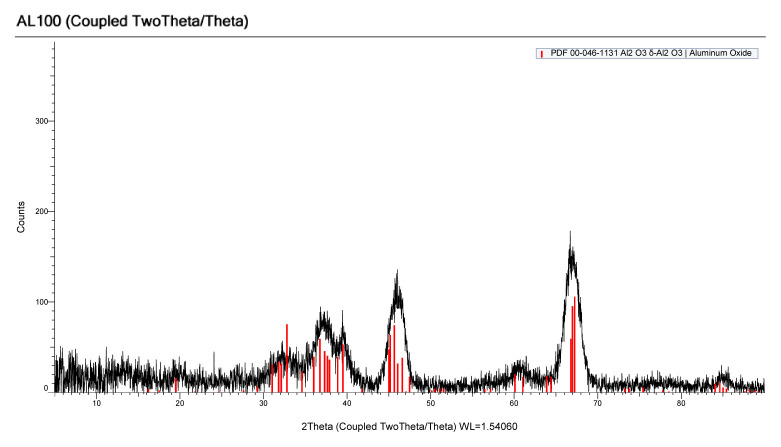
XRD pattern of Al_2_O_3_.

Figure 1 shows the XRD pattern of natural zeolite. According to the standard cards number 00-012-0275 and 00-062-0761 main peaks correspond to erionite and chabazite-Mg, respectively. According to the reference this Mexican zeolite contains 46% of erionite and 54% of chabazite and Si/Al ratio is 3.7 [18]. Figure 2 shows XRD pattern of alumina (Al_2_O_3_), where four main broad peaks at 32.8º, 36.7º, 45.6º, and 67.2º with standard card number 00-046-1131 were clearly observed. Alumina particle size was calculated using the Scherrer equation [19] for major peak 67.2° and found to be 4.6 nm. Alumina nanoparticles are recognized as promoting solar light induced photocatalyst due to reduce the band gap [10].

### 3.2. SEM

**Figure 3:**
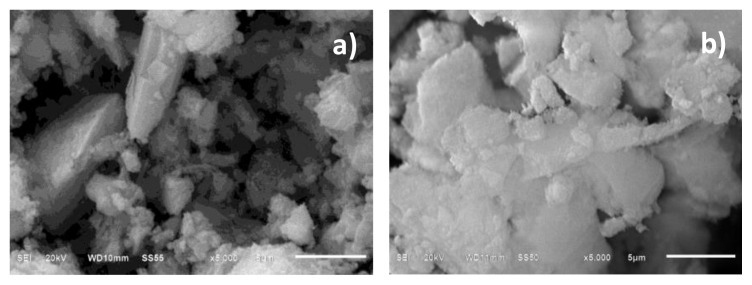
Scanning electron micrographs of a) erionite and b) Al_2_O_3_ at 5000 amplifications.

Figure 3 shows erionite and alumina (Al_2_O_3_) micrographs at 5000 amplifications. For erionite some border particles well defined is observed with morphology of elongated prisms as reported in the literature [20], the rest of the particles do not show a defined shape and are presented in aggregates with particles of different sizes. In the case of alumina, larger particles appear to be covered with small particles, and they do not have uniform shapes or sizes. Sol-gel process promotes amorphous alumina particles [21] and has been widely used to obtain micro- and mesoporous materials with homogeneous composition and controlled porosity.

### 3.3. EDS

**Figure 4:**
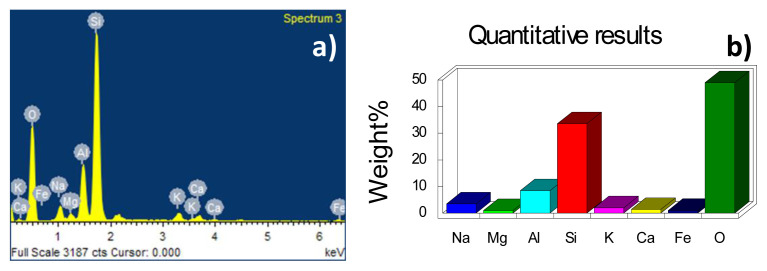
(a) Spectrum of energy dispersive spectroscopy and (b) elemental chemical composition in weight percentage of erionite.

**Figure 5:**
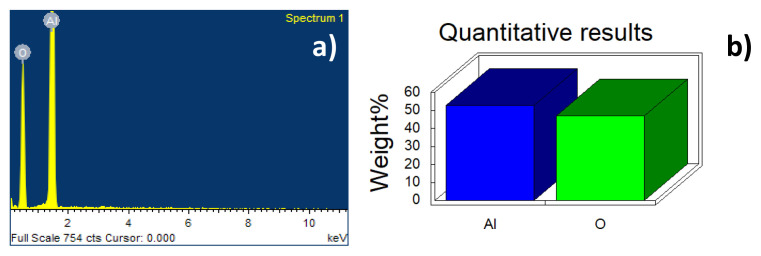
(a) Spectrum of energy dispersive spectroscopy and (b) elemental chemical composition in weight percentage of Al_2_O_3_.

The composition of both catalysts was determined using EDS analysis (Table 1) and for erionite the presence of seven cations is confirmed where silicon and aluminum represent the highest proportion (Figure 4) with a Si/Al ratio of 3.9 similar as reported in the literature 3.7 [18]. The alumina synthesized by the sol-gel method presents purity according to what is observed in the EDS spectrum (Figure 5) because there are no impurities after the synthesis process.

### 3.4. Physicochemical characterization of the textile wastewater

The physicochemical characterization of the textile wastewater before applying the photochemical and microbiological treatments was carried out to determine the initial conditions of the effluent when the sample was taken. After applying both treatments, the physicochemical parameters were measured again to compare them with the initial values and to evaluate the efficiency of the combined process in the degradation of pollutants (Table 2). Textile wastewater is characterized by high values of parameters as conductivity, color and COD according reported in literature [22,23].

Physicochemical parameters were compared with Mexican regulations applied to river discharge; pH was slightly reduced after the combined treatments remaining in established limits. Total suspended solids (TSS) were reduced considerably after both treatments, but accomplishment for agricultural irrigation limits is higher than the limits for public use after combined treatments with erionite. Finally, TSS is lesser established limits for protection aquatic life after the combined treatments with alumina.

Parameters as conductivity, COD, turbidity, and color are not specified in Mexican regulations. However, these parameters are associated with water quality and were diminished after photocatalytic and microbiological processes. PA+BC1 reached a COD reduction of 85%, PE+BC1 of 65.1%, PA+BC2 of 92% and PE+BC2 of 68.6%. In the case of COD, values higher than 200 mg/L are an indicator of water stronger polluted established by National Water Commission in Mexico and the best combined treatment photocatalysis with alumina and especially with consortium 2 (PA+BC2) reduced COD below this limit. Decolorization was reached 86.4% with PA+BC1, 68.2% with PE+BC1, 91.2 % with PA+BC2, and 714.3% with PE+BC2.

### 3.5. Photocatalytic and biological treatments of the textile wastewater

**Figure 6:**
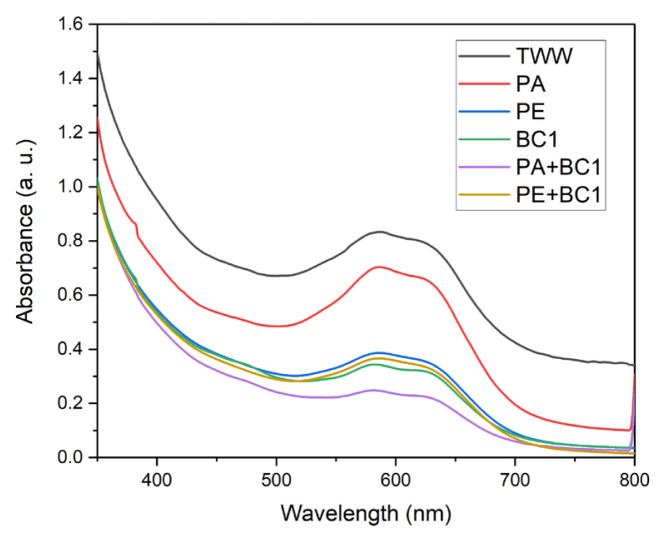
UV-Vis spectra of a) textile wastewater (TWW), b) photodegradation test with Al_2_O_3_ catalyst (PA), c) photodegradation test with erionite catalyst (PE), d) microbiological test with consortium 1 (BC1), e) microbiological test with consortium 1 after photodegradation test with Al_2_O_3_ catalyst (PA+BC1), and f) microbiological test with consortium 1 after photodegradation test with erionite catalyst (PE+BC1).

Figure 6 shows UV-Vis spectra of textile wastewater and photocatalytic, microbiological and combined treatments with microbial consortium 1 (BC1). A broad absorption band for textile wastewater is observed between 500 nm and 700 nm, it was decreased with every treatment. In the case of photocatalytic processes with Al_2_O_3_ (PA) and erionite (PE), spectra show that erionite catalyst is more effective than Al_2_O_3_, reached dye degradation 54.75% and 28.62%, respectively (Table 3). For treatment with microbial consortium 1 (BC1), spectrum shows a greater degradation than photocatalysis reached to 56.93%. However, when treatments are applied sequentially (PA+BC1 and PE+BC1) the degradation of the textile wastewater increases. In the combination of the photocatalysis with the erionite catalyst and the microbial consortium 1 (PE+BC1), a slight improvement in biodegradation is seen (57.65%), meanwhile the greater dye degradation was with photocatalysis having Al_2_O_3_ as a catalyst and microbial consortium 1 (PA+BC1) 64.16% (Table 3).

**Figure 7:**
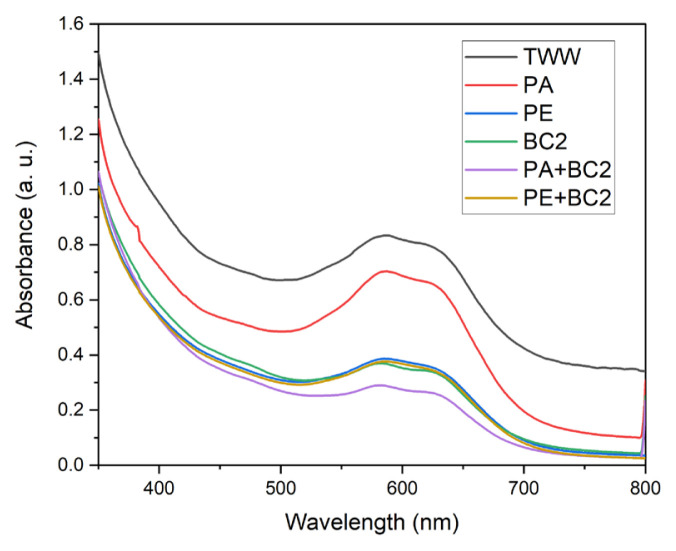
UV-Vis spectra of a) textile wastewater (TWW), b) photodegradation test with Al_2_O_3_ catalyst (PA), c) photodegradation test with erionite catalyst (PE), d) microbiological test with consortium 2 (BC2), e) microbiological test with consortium 2 after photodegradation test with Al_2_O_3_ catalyst (PA+BC2), and f) microbiological test with consortium 2 after photodegradation test with erionite catalyst (PE+BC2).

**Figure 8:**
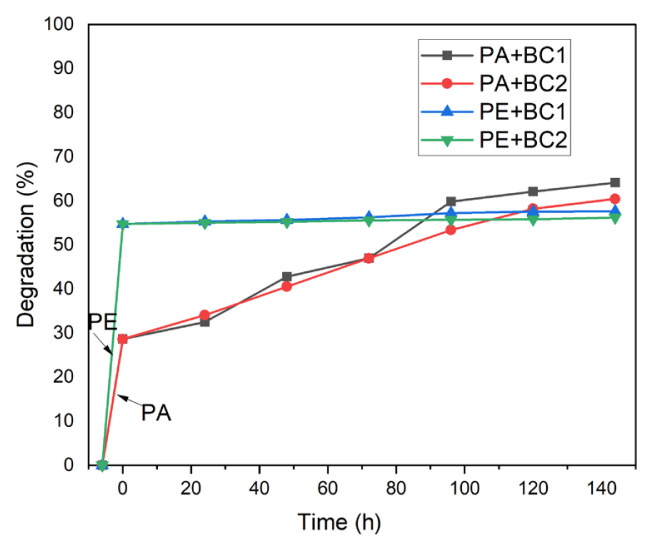
Percentages of degradation of pollutants presents in textile wastewater, initially with photocatalytic processes by 6 h using natural erionite zeolite (PE) and aluminum oxide (PA), followed by microbiological treatment by 6 days with consortium 1 (BC1) and consortium 2 (BC2).

Figure 7 shows UV-Vis spectra of textile wastewater and photocatalytic, microbiological and combined treatments with microbial consortium 2 (BC2). In this case, spectrum of BC2 shows a greater degradation than photocatalysis with Al_2_O_3_ reached to 53.56% but is lower than degradation with BC1 (56.93%). However, when treatments are applied sequentially(PA+BC2 and PE+BC2) the degradation of the textile wastewater increases. In the combination of the photocatalysis with the erionite catalyst and the microbial consortium 2 (PE+BC2), a slight improvement in biodegradation is seen (56.20%), meanwhile the greater dye reduction was with photocatalysis having Al_2_O_3_ as a catalyst and microbial consortium 2 (PA+BC2) reached to 60.44% dye degradation (Table 3). Despite this, PA+BC2 treatment manages to considerably reduce the chemical oxygen demand below 200 mg/L and total suspended solids, conductivity, turbidity, and color. The foregoing contributes to reducing the number of contaminants present in textile wastewater and allows compliance with the maximum permitted limits established in the official Mexican regulations for this type of discharge. Photocatalysis with alumina promotes higher degradation of pollutants than erionite. Meanwhile, microbial consortium 1 (*Escherichia coli N16, Serratia k120, Pseudomonas putida B03 and Enterobacter hormaechei*) allows further degradation than microbial consortium 2 *(Escherichia coli N16, Serratia Mc107, Enterobacter N9, Enterobacter hormaechei Mc9*). From the UV-Vis absorption spectra, the percentage of degradation of the pollutants present in the textile wastewater was determined, based on the area under the curve of each spectrum and normalizing the data (Table 3). Figure 8 compares the percentages of degradation of the four combined sequential processes resulting PA+BC1 > PA+BC2 > PE+BC1 > PE+BC2, considering that textile wastewater has 0% degradation of the pollutants.

Finally, kinetic parameters were determined to state the degree of reactions (Table 4). In all combined processes, the kinetic constants were first order if the reactions depend on dye concentration present in textile wastewater.

## 4. Discussion

The individual photocatalytic and microbiological processes are not as efficient if they are used individually for the transformation of recalcitrant pollutants or take them to a mineralization process, therefore combined processes are currently being studied that can increase this efficiency and above all eliminate the toxicity of some intermediate compounds. A combination of photocatalysis and biodegradation is a promising approach to the removal of xenobiotic organic compounds from wastewater, as photocatalysis splits molecules into simpler intermediates through attack by free radicals (hydroxyl radical (•OH), superoxide anions (O_2_•) and hydrogen peroxide (H_2_O_2_)) and generates easily biodegradable products that are then mineralized by microorganisms [24,25].

The biodegradation time of dyes by microorganisms depends on the growth rate, environmental conditions and is directly related to the rate at which the waste metabolizes, due to the above, removing the biodegradation kinetics is essential for efficient design of reactors in the treatment of dye effluents [22].

In natural erionite zeolite, microporous porosity is predominant with volume calculated around 0.11 cm^3^/g, and its BET surface area was reported 303.4 m^2^/g [20]. An important research area of applications for Mexican erionite is the retention of cations such as cobalt, cadmium, and thorium from aqueous solutions due to its cation exchange capacity higher than other zeolites. Sorption capacity of chemical species in Mexican erionite depends on the type of organo-aqueous media as reported in a study of mercury removal [26]. In this case, microporous porosity, high surface area and cation exchange capacity of natural zeolite erionite used for photocatalytic tests could be responsible for dye discoloration after 6 h of solar radiation exposition. Pollutant molecules can be adsorbed to surface area and promote chemical bond breaking.

Alumina used in photocatalytic processes with a semiconductor as doped titania results in reducing the band gap of titania which promote its photocatalytic activity in the visible spectrum [27]. Several studies have been directed to determine the band gap of mesoporous alumina showing a large spectral range for optical applications such as narrowband filters [28]. In this case, alumina represents an important potential for photocatalytic processes with solar radiation [29].

An aspect to highlight in this research is the ratio of catalyst to volume of treated water, which is 45 mg to 500 mL, resulting in a relatively low concentration of catalyst 0.09 g/L that allows remove some color of textile wastewater. While in other investigations that also use sunlight, they use high catalyst ratios of 2 g/L [30] reaching total mineralization of a blue dye in 60 min. They also use synthetic colored water, unlike real textile wastewater that contains varied components and in unknown concentrations.

The water resulting from the combined photocatalysis-microbiological degradation processes was tested for toxicity using Daphnia magna essay, obtaining that none of the effluents shows toxicity for the analysis of the LD50.

Decoloring real textile wastewater was reached in photocatalytic processes with solar radiation and two materials used as catalysts, alumina synthesized by sol gel method and natural Mexican zeolite erionite. The last one showed a higher color degradation, diminish initial concentration to 45.25%. It can be attributed to textural parameters of the erionite, which provided catalytic sites for colorants molecules and its fragments meanwhile those are broken by solar radiation. Biodegradation test after photocatalytic processes increases decoloring considerably when alumina was used. However, no great difference was observed in photocatalytic processes with erionite. Maybe color molecules and its fragments were trapped in erionite microporous structure and cannot be available for consumption of bacteria’s. Finally, the combined processes photocatalysis with alumina and further biodegradation with consortium 1 (*Escherichia coli* N16, *Serratia* k120, *Pseudomonas putida* B03 and *Enterobacter hormaechei*) showed the higher degradation of color in real textile wastewater lowering it to 35.84%.

Additionally, combined processes promoted reduction of the principal physicochemical parameters related to water quality and allows compliance with the maximum permissible limits established by current Mexican regulations for the discharge of wastewater to national surface water.

**Figure 9:**
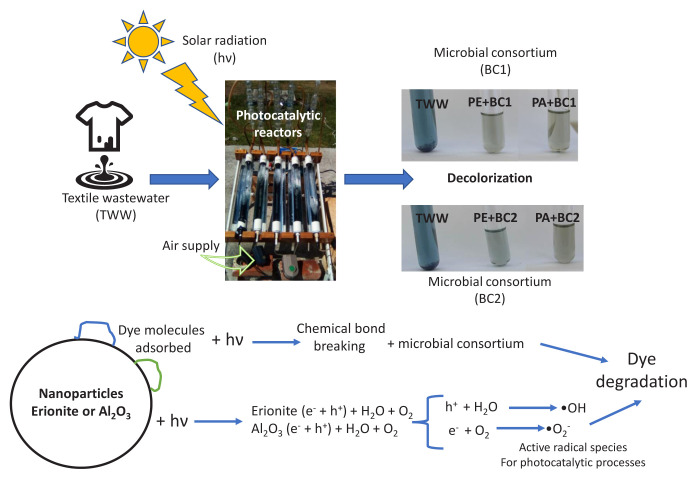
Schematic representation of the proposal combined treatment, considering two routes for dye degradation in textile wastewater: adsorption and photocatalytic reactions.

Figure 9 showed a schematic representation of the proposal combined treatment, considering two routes for dye degradation. Adsorption of dye molecules on surface area of erionite and alumina, with solar radiation chemical bond breaking is promoted and more biodegradable compounds are formed. Photocatalytic by electron-hole formation on material surfaces generating the formation of radicals highly oxidants.

## 5. Conclusion

The combined photocatalytic and microbiological processes are effective for the elimination of recalcitrant compounds in wastewater such as textile dyes, being an important alternative since they are friendly to the environment by using solar energy and remove the dyes not only change the place of contamination such as physicochemical processes. In this work, photocatalysis with alumina followed by a microbiological process showed the best results diminishing two parameters related to water quality as COD and color, higher than 90%. In addition, the wastewater treated by the sequential process complies with the limits established in the Mexican regulation so that the industries can discharge it without penalty and without causing a negative impact to the receiving water bodies. Finally, there is no toxicity in the treated water, therefore it has the great possibility of being reused for services that do not have direct contact with people.

## Figures and Tables

**Table 1:** Elemental chemical composition expressed in weight percentage of erionite and Al_2_O_3_ obtained by EDS.

Element	Na	Mg	Al	Si	K	Ca	Fe	O	Total
Erionite	3.43	1.10	8.36	33.42	2.14	1.52	1.20	48.82	100.00
Al_2_O_3_	-	-	52.93	-	-	-	-	47.07	100.00

it does not contain.

**Table 2:** Physicochemical parameters measurements before and after the combined treatments.

Parameter analyzed	Before treatment	After the combined treatment				Mexican Regulations NOM-001-SEMARNAT-1996		
	Textile WW	Consortium 1		Consortium 2		Rivers		
		Al_2_O_3_ catalyst (PA+BC1)	Erionite catalyst (PE+BC1)	Al_2_O_3_ catalyst (PA+BC2)	Erionite catalyst (PE+BC2)	Agricultural Irrigation	Public use	Protection aquatic life
pH	7.89	7.2	7.4	7.1	7.4	5–10	5–10	5–10
Total suspended solids (mg/L)	525.64	35.45	165.48	19.45	135.89	200 mg/L	125 mg/L	60 mg/L
Conductivity (mS cm^−1^)	68.35	6.45	45.78	6.25	23.78	N.S	N.S	N.S
COD (mg/L)	1872	289	654	150	589	N.S	N.S	N.S
Turbidity (NFU)	352	41	91	26	89	N.S	N.S	N.S
Color (m^−1^)	47.6	6.5	15.14	4.2	13.65	N.S	N.S	N.S

NFU = nephelometric units.

N.S = not specified.

**Table 3:** Degradation values expressed as percentage, of each simple and the combined treatment.

					Consortium 1		Consortium 2	
Textile wastewater	Photocatalysis with Al_2_O_3_	Photocatalysis with Erionite	Microbiological test	Microbiological test	Al_2_O_3_ catalyst	Erionite catalyst	Al_2_O_3_ catalyst	Erionite catalyst
TWW	**PA**	**PE**	**BC1**	**BC2**	**PA + BC1**	**PE + BC1**	**PA + BC2**	**PE + BC2**
% Degradation	28.62	54.75	56.93	53.56	64.16	57.65	60.44	56.20

**Table 4:** Kinetic parameters obtained for sequential processes: photocatalytic and microbiological.

	Consortium 1		Consortium 2	
	Al_2_O_3_ catalyst	Erionite catalyst	Al_2_O_3_ catalyst	Erionite catalyst
	PA + BC1	PE + BC1	PA + BC2	PE + BC2
	Cero degree			
R^2^	0.960172	0.964960	0.986989	0.979175
	First degree			
R^2^	0.963149	0.964962	0.992911	0.979219
K_1_ (min^−1^)	0.004782	0.000458	0.004096	0.000225
	Second degree			
R^2^	0.956130	0.964859	0.988034	0.979212
